# A segmental osteotomy with 3D virtual planning to move a malpositioned dental implant

**DOI:** 10.1186/s40729-021-00359-2

**Published:** 2021-08-12

**Authors:** Dario Andrés Bastidas, Lina Roldan, Pamela Ramirez, Andrés Munera

**Affiliations:** 1 Department of Maxillofacial Surgery, University CES, Medellin, Colombia; 2 Department of Orthodontic, University CES, Medellin, Colombia; 3 Department of Prosthodontics, University CES, Medellin, Colombia

**Keywords:** Dental implant, Anterior segmental osteotomy, 3D planning, Computer-aided design (CAD), Computer-aided manufacturing (CAM)

## Abstract

**Background:**

Correct positioning and alignment of dental implants are crucial to successfully meet the aesthetic and functional criteria in implant-prosthetic rehabilitation. When an implant is in the wrong position, especially in the esthetical zone, there are limited options to solve it. Some techniques have been described to reposition implants, such as reverse torque, trephine drills, and segmental osteotomies; current approaches aim to reduce the damage of the periimplant tissues.

**Case presentation:**

A 20-year-old man with good general health was referred to the oral and maxillofacial surgery department of the CES University, Medellin Colombia in 2017, He had undergone a previous camouflage orthodontic therapy for a dental Class III, which finished in 2014, posteriorly a dental implant was placed in 2015 to replace upper right lateral incisor (1.2) before vertical growth of maxilla was complete; therefore, the implant was retained in a coronal position. A segmental osteotomy was suggested to reposition the implant in a more caudal position, a 3D surgical cut guide obtained by virtual planning was used to increase osteotomy accuracy and lower human error, to avoid the risk of damaging the adjacent tissues and to achieve a predictable result.

**Conclusions:**

The segmental alveolar osteotomy is an effective alternative to reposition an implant; however, it must be carefully planned because human error remains a possibility that may affect the final result. Therefore, 3D planning is a better way to minimize these mistakes during the surgical procedure and the final position of the implant.

## Introduction

Correct positioning and alignment of dental implants are crucial factors in achieving satisfactory aesthetic and functional results. However, some implants, for many reasons, may be mispositioned, or sometimes in growing patients at early stages of life. To correct that position, there are several options, such as prosthetic compensation, replacement, or transfer of the position of the implant [1, 2], and if prosthetic corrections are not sufficient, the malposition implant can be left alone under the soft tissue or submerged completely into the bone. Another option is to remove the implant [3, 4]. However, in the anterior zone of the maxilla known as the “aesthetic zone” implant removal frequently results in hard and soft tissue defects requiring corrections with advanced tissue regeneration procedures before inserting a new implant [5].

Dental implants with the wrong position have been treated through segmental alveolar osteotomies [6, 7]. This technique had been initially used to treat ankylosed maxillary canines and to close one-tooth diastemas [8]. It has been described in some case reports in the literature with some variations to each technique. The basic concept is to move a bone-implant block, which is immediately stabilized in the new position by rigid fixation [6, 7, 9–20]. Besides, this bone-implant block could have a gradual movement if applying orthodontic forces or protocols of distraction osteogenesis [15, 17, 21, 22].

When the patient is still growing, there is an inferior displacement of the anterior maxilla approximately 1.2 mm/year, resulting in disharmony of oral implants in the anterior zone when compared to the natural teeth final position. On the other hand, during adolescence, the maxillary molars and incisors erupt 1.2 to 0.9 mm/year [23]. Those factors must be considered when evaluating the possibility to use dental implants at early ages. Vertical growth of the maxilla continues until at 17 or 18 years for girls and even later for boys. However, between the ages of 15 to 25, the teeth can move an amount of 5 mm vertically [24].

## Case report

**Fig. 1:**
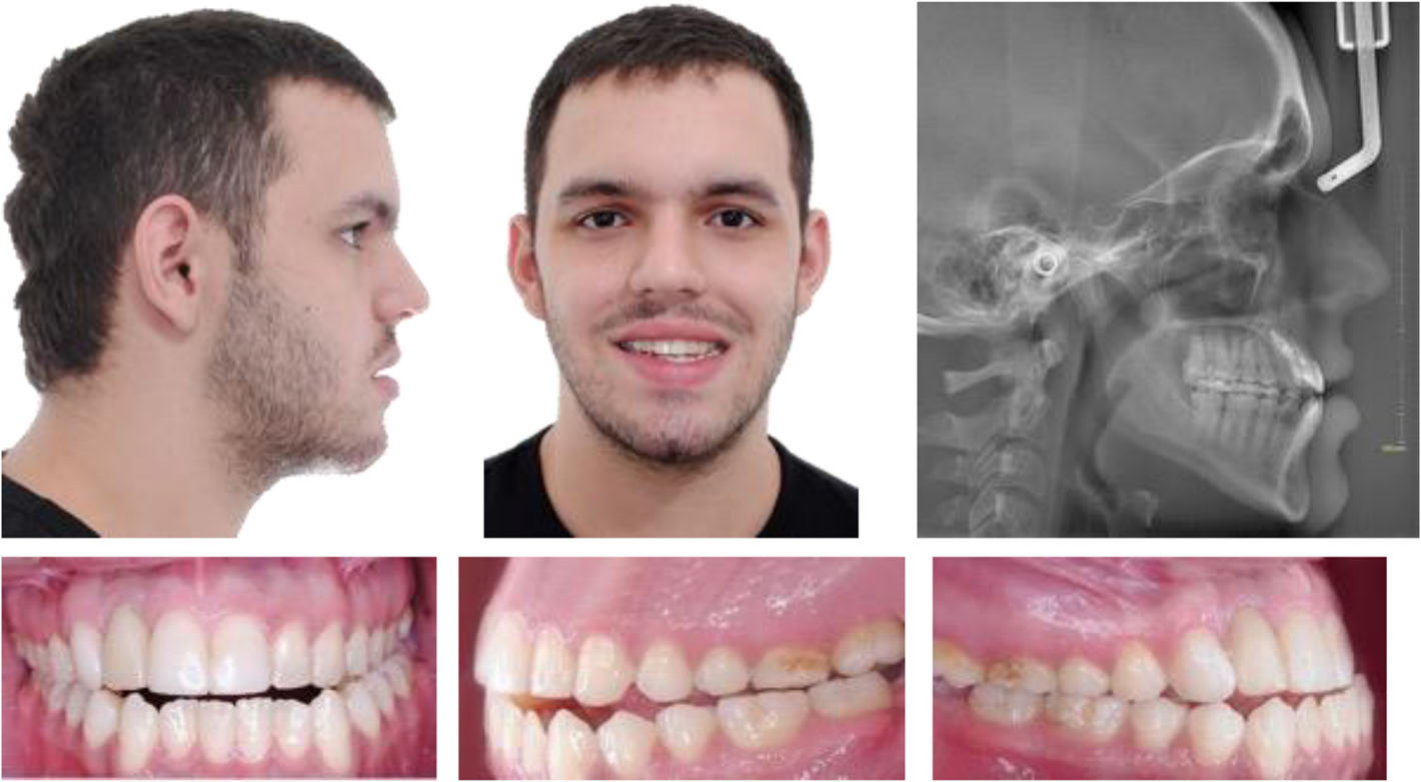
Pre-treatment extra-oral and intra-oral photos, and lateral cephalic X-ray

A 20-year-old man with good general health was referred to the oral and maxillofacial surgery department of the CES University, Medellin, Colombia in 2017, He had undergone a previous camouflage orthodontic therapy for a dental Class III, which finished in 2014, posteriorly the patient had a dentoalveolar trauma resulting in avulsion of the upper right lateral incisor (1.2) and required a dental implant in 2015 to replace it. Those therapies were done in another institution; therefore all of the information on the previous procedures and supplies used was not fully known. Nonetheless, it was possible to recover some information on the implant, it was a tapered implant with 3.5 mm diameter and 12 mm length and internal hexagonal conexion. On clinical examination, the patient presented with a skeletal and dental Class III due to maxillary hypoplasia and a prognathic mandible, anterior crossbite and open bite, lingualized lower incisors, the mentioned dental implant, and multiple attritions in the molars occlusal surface (Fig. 1).

**Fig. 2:**
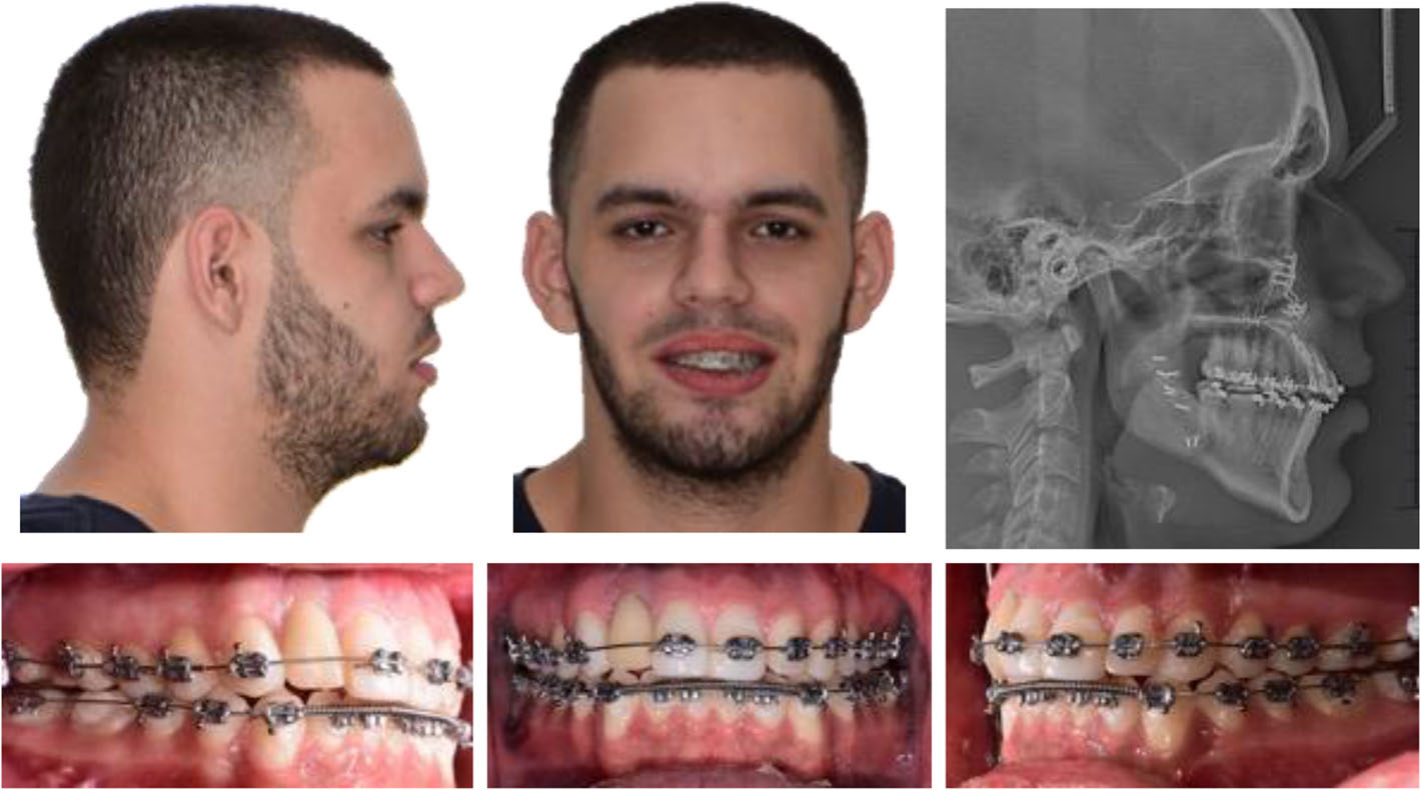
Extra-oral and intra-oral photos, and lateral cephalic X-ray during treatment

The treatment plan suggested for this patient was orthognathic surgery with surgery first protocol, orthodontic treatment, and a segmental osteotomy to move the dental implant to a caudal position once the orthodontic treatment was finished; the occlusal surfaces of molars and premolars were restored to get aesthetic-functional rehabilitation of the occlusal plane using posterior resin (P70-3M™ resin). Orthodontic treatment was performed with conventional 0.018 × 0.025 inches braces. Leveling and alignment began with 0.014 inches nickel-titanium arch-wire, followed by 0.016, 0.016 × 0.016. During the first arch-wires, anteroinferior teeth were not included in the arch. The occlusal plane was restored, and then 0.016 × 0.016 inches nickel-titanium arch-wires were placed, followed by 0.016 × 0.022, finally, stainless steel 0.016 × 0.022 inches rectangular arch-wires were used for the orthodontic finishing stage. The upper right lateral incisor (1.2 implant) was never included in the orthodontic treatment (Fig. 2).

**Fig. 3:**
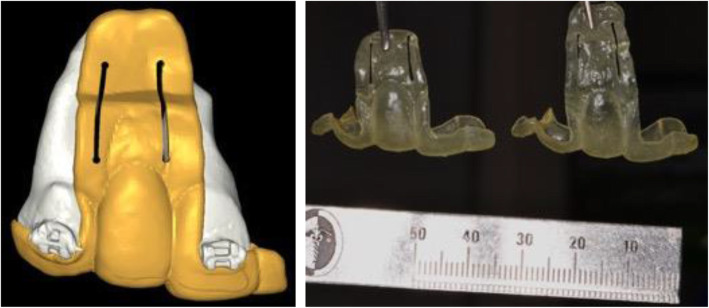
Surgical guide for segmentary osteotomy

When the orthodontic treatment was finished, 3D planning for the alveolar osteotomy to reposition the implant was performed. Digital Imaging and Communication on Medicine (DICOM) and Standard Triangle Language (STL) were used to create the surgical guide, and those files were processed in a 3D software (NemoStudio 2018 version, Spain) to build a surgical guide, which had 3 cuts, on the top, mesial, and distal areas of the implant (Fig. 3).

## Surgical procedure

**Fig. 4:**
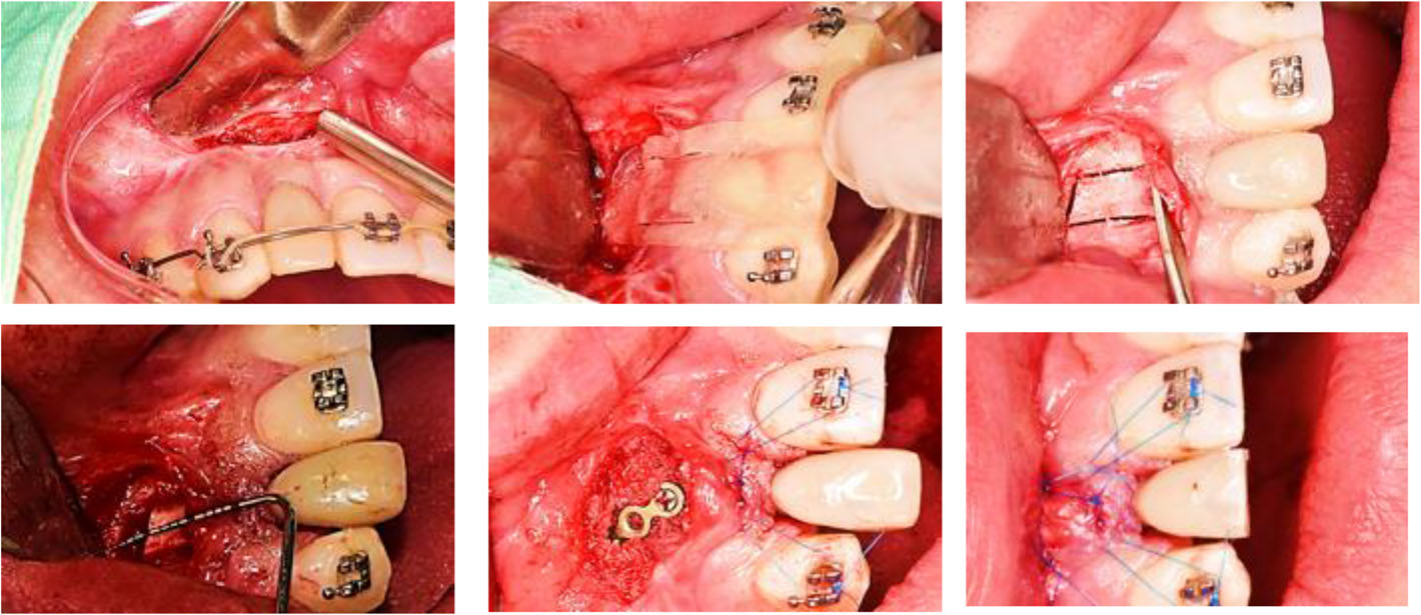
Surgical procedure

It was performed under local anesthesia (Lidocaine 2% with epinephrine 1:80.000). The access was achieved with a horizontal incision, coronal to the mucogingival line to maintain the marginal gingival harmony, tunnel dissection down to the alveolar crest, and upper to the nasal notch. After stabilization of the surgical guide with the contiguous teeth, the osteotomy was performed using Piezotome tips of 0.1 mm thickness (Piezotome Solo LED, Acteon, France, Tip reference 602990/1 and 602965/1), aiming to preserve the palatal soft tissue and allow it to join the coronal movement of the bone segment; the osteotomy was then finished with osteotomes. The ceramic crown was cut and polished until a similar height to the contralateral incisor was achieved, the osteotomy segment was mobilized, and the new position was stabilized with a miniplate and a screw of 1.2 mm osteosynthesis system (Mondeal). The osteotomy´s gap was 7 mm, and it was filled in with pulverized bone graft (Puros® Cancellous Particulate Allograft). A connective tissue graft was placed under the keratinized gum, and it was fixed to the orthodontic arch-wire using suture suspension in order to improve the thickness of the gingiva. Closing of the wound was done with non-absorbable monofilament nylon 6.0 (Fig. 4).

**Fig. 5:**
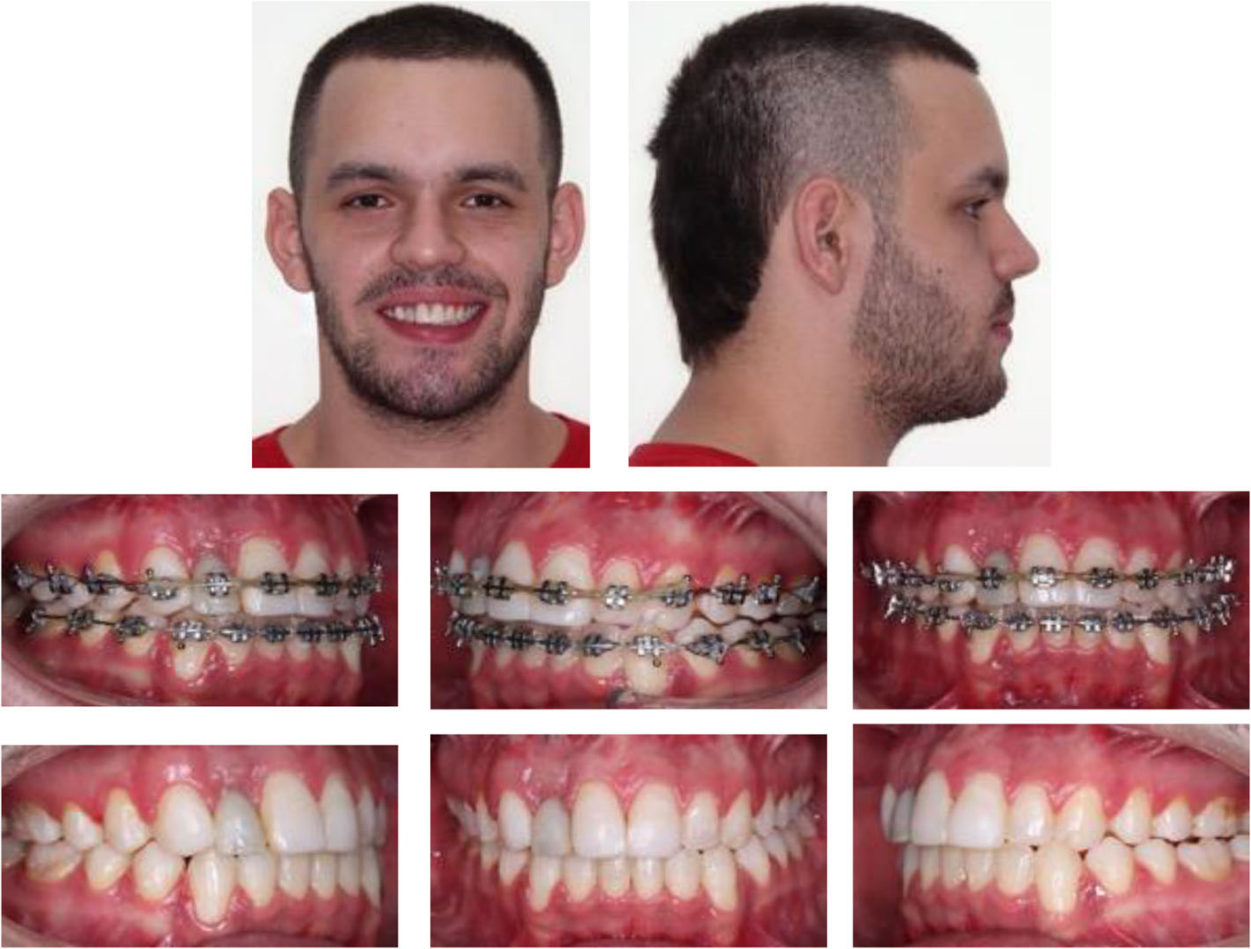
Finishing stage of orthodontic treatment

Finally, after 4 weeks of the surgical procedure, a passive bracket was adhered on the buccal surface of the 1.2 implant crown, and the finishing stage of the orthodontic treatment started 2 weeks after that. The braces were removed 6 months later to allow the gum and the bone regeneration to stabilize; at this point, the zenith of the upper right lateral incisor was at the same level as the contralateral incisor (Fig. 5). Once this treatment was finished, the patient was redirected to the prosthodontics department to change the definitive crown restoration.

## Discussion

Dental implant in the aesthetic area can be tricky, and there are several factors, such as lack of surgical planning, surgical guide inaccuracies, inexperience, and wrong placement in the insertion of the implant, all of which can result in osseointegrated implants placed in non-optimal positions that lead to poor prosthetic restoration. These factors must be evaluated before treatment to aim for a successful result [25, 26]. This matter could also affect growing patients since the vertical growth of the maxilla continues up until 25 years [23, 24], because of that, clinicians ought not to put osteointegration implants before this age. One alternative to the growing patient to maintain bone level and allow the use of a temporary fixed crown is mini-screws [27].

When the malposition of a dental implant is considered mild, it can be corrected by prosthetic modifications, such as the use of custom-made abutments, angled implant abutments, and methods of cementation of the prosthetic device rather than screw systems; however, when malposition is severe, it is necessary to perform an intervention directly on the implant [12, 19]. Removal of an osseointegrated implant usually requires invasive surgery and damage to the surrounding bone; Roy et al. reported in 2020 [5], the principal reasons for implant removal are periimplantitis and crestal bone loss, mispositioning only accounts for a 8.4%; some commonly used approaches for implant removal are reverse torque and trephine drills, reverse torque being not only the principal but also the most conservative approach requiring removal of little to no bone, trephine drills were indicated when torque values exceeded 200 N/cm; however, some of the reverse torque techniques described the need to use a trephine drill in the coronal aspect of the implant to ease the explantation, in addition, the success rate for reverse torque was only of 87.7%; it is also important to address immediate implant placement, in the mentioned study it was only possible in 22.3% of the cases among all presented techniques [5]. Due to the unpredictability of these procedures on the amount of bone left after surgery, some patients must undergo several surgical procedures that may often be necessary to complete a satisfactory result, such as bone a soft tissue augmentation before considering a new implant placement; also, all these procedures can present high costs for the patient and lengthen in total treatment time [5]. Segmental osteotomy has been described in some case reports as an additional alternative to relocate a mispositioned implant without the need to remove the implant itself, aiming for preservation of already achieved osteointegration and bone architecture and to lower the risk to require additional procedures before new implant positioning can be done [3, 6, 7, 9–14, 16–20, 22]. Nowadays, technology is an ally, making possible to plan all types of osteotomies which minimize error during surgical procedure; besides, the new implant position can also be planned to achieve a proper restoration. Stacchi et al. reported a case back in 2012 [12] using CBCT for a segmental osteotomy planning, from which he obtained a stereolithographic model of the maxillary bone and was then replicated in a stone cast model were the surgery was manually planned to further design a titanium guide to reposition the bone block including the implant; nonetheless, the osteotomies were not virtually planned and this can still provide some level of error during surgical procedure that can cause failure of the repositioning, which is why, in this paper, a surgical guide for the osteotomies was designed using 3D virtual planning, in order to reduce human error [12].

There are similar principles in cases reported in the literature, such as the vestibular approach always be the election of incision, preservation of the palatal or lingual vascular supply, care to avoid damage to adjacent natural teeth, and rigid fixation of the bone block [7]. Stacchi et al. suggested three fundamental factors to alveolar osteotomy for implants, maximum preservation of blood supply during the early phases of healing, minimum gap between the mobilized block and adjacent bone, firm and stable bone block fixation [7]. On the other hand, the absence of micromovements is a fundamental element in promoting osseous repair, because the absence of stability of the osteotomized segment can result in non-union or malunion of the bone fragment [28, 29]. The issue is the grand amount of differences in described techniques, from the osteotomy planning to the relocation of the bone segment; some authors even consider the approach of a distraction osteogenesis driven by orthodontic treatment, thus making difficult to unify techniques and therefore to analyze success rate and best approach.

## Conclusions

The segmental alveolar osteotomy is an effective alternative to reposition an implant; however, it must be carefully planned because human error remains a possibility that may alter the final result. Therefore, 3D planning is a better way to minimize these mistakes during the surgical procedure and final position of the implant. More studies, greater patient samples, and longer follow-up time are needed to define the technique that can lead to a higher success rate and reduce damage to periimplantar tissues.

## Data Availability

Not applicable.

## Abbreviations

DICOM: Digital Imaging and Communication on Medicine
STL: Standard Triangle Language
